# Light-Responsive and Antibacterial Graphenic Materials as a Holistic Approach to Tissue Engineering

**DOI:** 10.1021/acsnanoscienceau.4c00006

**Published:** 2024-06-07

**Authors:** Andrea Ferreras, Ana Matesanz, Jabier Mendizabal, Koldo Artola, Yuta Nishina, Pablo Acedo, José L. Jorcano, Amalia Ruiz, Giacomo Reina, Cristina Martín

**Affiliations:** † Department of Bioengineering, Universidad Carlos III de Madrid, Leganés 28911, Spain; ‡ Department of Electronic Technology, Universidad Carlos III de Madrid, Leganés 28911, Spain; § Domotek ingeniería prototipado y formación S.L., San Sebastián 20003, Spain; ∥ Graduate School of Natural Science and Technology, Okayama University, Okayama 700-8530, Japan; ⊥ Research Core for Interdisciplinary Sciences, Okayama University, Okayama 700-8530, Japan; # Instituto de Investigación Sanitaria Gregorio Marañón, Madrid 28007, Spain; ∇ Institute of Cancer Therapeutics, School of Pharmacy and Medical Sciences, Faculty of Life Sciences, University of Bradford, Bradford BD7 1DP, United Kingdom; ○ Empa Swiss Federal Laboratories for Materials Science and Technology, St. Gallen 9014, Switzerland

**Keywords:** photothermal therapy, graphene derivatives, 4D bioprinting, alginate, tissue engineering

## Abstract

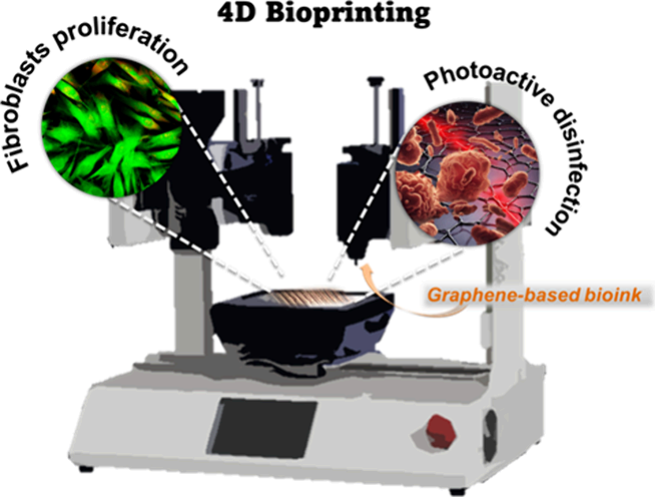

While the continuous development of advanced bioprinting technologies is under fervent study, enhancing the regenerative potential of hydrogel-based constructs using external stimuli for wound dressing has yet to be tackled. Fibroblasts play a significant role in wound healing and tissue implants at different stages, including extracellular matrix production, collagen synthesis, and wound and tissue remodeling. This study explores the synergistic interplay between photothermal activity and nanomaterial-mediated cell proliferation. The use of different graphene-based materials (GBM) in the development of photoactive bioinks is investigated. In particular, we report the creation of a skin-inspired dressing for wound healing and regenerative medicine. Three distinct GBM, namely, graphene oxide (GO), reduced graphene oxide (rGO), and graphene platelets (GP), were rigorously characterized, and their photothermal capabilities were elucidated. Our investigations revealed that rGO exhibited the highest photothermal efficiency and antibacterial properties when irradiated, even at a concentration as low as 0.05 mg/mL, without compromising human fibroblast viability. Alginate-based bioinks alongside human fibroblasts were employed for the bioprinting with rGO. The scaffold did not affect the survival of fibroblasts for 3 days after bioprinting, as cell viability was not affected. Remarkably, the inclusion of rGO did not compromise the printability of the hydrogel, ensuring the successful fabrication of complex constructs. Furthermore, the presence of rGO in the final scaffold continued to provide the benefits of photothermal antimicrobial therapy without detrimentally affecting fibroblast growth. This outcome underscores the potential of rGO-enhanced hydrogels in tissue engineering and regenerative medicine applications. Our findings hold promise for developing game-changer strategies in 4D bioprinting to create smart and functional tissue constructs with high fibroblast proliferation and promising therapeutic capabilities in drug delivery and bactericidal skin-inspired dressings.

## 1 Introduction

Hydrogel-based materials have gained prominence within the tissue engineering landscape due to their versatile mechanical properties and amenability to cell encapsulation. Yet, as we strive to push the boundaries of regenerative medicine, there is a growing imperative to augment the regenerative potential of these hydrogel constructs. In tissue engineering and regenerative medicine, bioprinting has emerged as a groundbreaking technology that precisely fabricates three-dimensional (3D) constructs, facilitating the generation of 3D structures by utilizing bioinks—biomaterials that encapsulate living cells.^1,2^ When the constructs are produced using “smart” materials capable of self-transformation into predefined shapes or adaptive responses to specific stimuli, they are categorized as “4D-printed materials”. In other words, the fourth dimension in 4D bioprinting refers to the temporal aspect, meaning that the printed structures exhibit dynamic behavior or undergo controlled transformations after fabrication: the 4D-printed structures or materials can change their shape, properties, or function in response to external stimuli such as temperature, light, pH, or specific biological signals.^3^

While certain traditional methods, such as solvent casting or melt molding, can generate porous scaffolds of diverse shapes and structures with commendable mechanical characteristics, they encounter challenges in attaining consistent micro- and macroscale pore distributions and reproducibility due to constraints inherent in their manufacturing processes.^4^ Moreover, in the past decade, research in the biofabrication field has grown exponentially to create relatively large, centimeter-scale objects.^5,6^ 3D bioprinting technologies emerge as an alternative to these limitations and can be categorized into three distinct process categories, namely, material jetting, vat photopolymerization, and material extrusion, according to the American Society of Testing and Materials (ASTM) standards.^7^ Material jetting involves depositing droplets of bioink layer by layer, offering high resolution and precision and making it suitable for printing intricate structures.^7^ However, the shear stress generated during droplet formation and deposition can affect cell viability, and cell deposition in inkjet-based bioprinting is still a challenge.^8^ Vat photopolymerization uses light to selectively solidify liquid photopolymerizable materials layer by layer.^9^ It provides high resolution and allows for the fabrication of complex geometries. However, exposure to UV light during printing can pose challenges for cell viability, as UV radiation may harm cells or interfere with bioink properties. Material extrusion involves the continuous deposition of bioink through a nozzle driven by pneumatic or mechanical forces.^4^ It offers versatility in using various bioink formulations, including those containing cells, growth factors, and biomaterials. Extrusion-based bioprinting typically exerts lower shear stress on cells than other techniques, contributing to better cell viability. Additionally, extrusion-based bioprinting systems are typically more affordable and easier to implement than other techniques.^10^ Due to these reasons, extrusion-based bioprinting is often considered the best choice. In this context, natural hydrogels based on gelatin, collagen, hyaluronic acid, fibrin, or alginate, among others, are highly used in 3D bioprinting due to their excellent biocompatibility, possible printability, and relatively low cost for tissue engineering.^11,12^ With 4D-printed structures, the ideal scaffold should exhibit a degradation behavior matching the regeneration process of the damaged tissue. The degradation of alginate hydrogels is a process that happens through an initial dehydration step, and it has been reported that for wound dressings, the hydrogel is degraded in the first week when used as a wound dressing agent in vivo, also stimulating collagen production in the injured area.^13^ Considering that skin infections have a higher risk of occurrence during the first week of the healing process,^14^ an alginate-based scaffold with biodegradable, biocompatible, and bactericidal functionalities would be an ideal material to design implants that will not require replacing wound dressings.

Fibroblasts are mesenchymal cells that play a pivotal role in epithelial–mesenchymal interactions ruling epidermal proliferation, differentiation, and formation of the extracellular matrix being integrated into a vast set of clinical applications, including the treatment of burns, chronic venous ulcers, and plastic surgery.^15^ Interestingly, fibroblasts have also recently been integrated into bioinks for 3D bioimprinting and tissue engineering applications.^16^ Montero et al. developed hydrogels composed of plasma-derived fibrin and thiolated hyaluronic acid. These gels allowed the maintenance of fibroblasts’ and keratinocytes’ normal proliferation levels, showing promising results for future applications in wound healing.^17^ More recently, we synthesized plasma-derived fibrin hydrogels containing the bactericidal GO/STREP hybrid for sustained antibiotic release, demonstrating significant potential for future fabrication of bactericidal skin equivalents for wound healing and treating extensively burnt patients.^18^ In a different study, a novel bioink made of plasma, alginate, and methylcellulose was presented as a promising material for several forms of bioprinted tissue equivalents; it proved to be exceptionally advantageous when combined with calcium phosphate cement, enhancing the biofabrication of bone-like constructs.^19^ However, finding a biocompatible matrix that can host fibroblasts and that can, per se, enhance cell proliferation is still challenging.

According to WHO, we are currently experiencing a silent pandemic due to the rapid spread of multidrug-resistant pathogens; it is estimated that no effective antibiotic will be available by 2050.^20^ Peri-implant infection is currently the most significant threat in clinics.^21^ Cell-containing scaffolds are much more difficult to disinfect than hard implants. Indeed, while hard implants can be easily pretreated (e.g., UV and ethylene oxide treatments), sterilizing cell-containing scaffolds, such as bioinks, could compromise cell functions. Current strategies for sterilizing cell-containing implants (e.g., skin transplant) rely on the massive use of antibiotics.^22^ Skin *Escherichia coli* infection point of care regards the use of a cocktail of antibiotics that includes ampicillin, tetracycline, and fluoroquinolones.^23^ However, it has been reported that the *E. coli* strains isolated from skin infections develop high virulence and resistance to most of the antibiotics, displaying high virulence like the one isolated from the catheters.^24^ It has also been reported that *E. coli* is the third-most prevalent isolated species, preceded solely by *Staphylococcus aureus* and *Pseudomonas aeruginosa*.^23^ Thus, alternative methods should be explored.

Photothermal therapy (PTT) is a promising technique for treating various diseases. In PTT, a photosensitizer (PS) converts light energy into local heat that can be used to modulate cellular behavior or induce cell ablation.^25−28^ A precise spatial and temporal control of the temperature is guaranteed by the external light stimulus applied. Additionally, novel PSs are developed to absorb in the near-infrared (NIR) region where soft human tissues are transparent. Thus, PTT displays high versatility where cellular functions such as proliferation, differentiation, and metabolic activity can be tuned by the PSs and the light source used.^29−32^ Due to its mechanism of action, PTT is also considered an elegant holistic antimicrobial strategy that does not induce resistance.^33^

Graphene-based materials (GBM) are a class of biocompatible and biodegradable carbon 2D nanomaterials with excellent photothermal and photodynamic performance.^34,35^ Graphene oxide (GO), for instance, has been reported to have a combined PTT/PDT effect when irradiated with NIR light, increasing its toxicity through heat generation and enhanced production of reactive oxygen species (ROS).^25^ The physicochemical properties of GBM, such as oxygen content, number of layers, or lateral size, strongly influence their interaction with light, dispersibility, biodegradation, and biosafety, thereby impacting their targeted applications.^36^

In this study, we describe the preparation of a bioink containing GBM for PTT. At first, we demonstrate that GBM display an effective bactericidal activity when irradiated with NIR light. Subsequently, we show that the bioinks containing GBM can be easily produced and processed. Finally, we found a promising synergy between photothermal activity and 3Dbioprinted materials to maintain fibroblast proliferation within the alginate-based hydrogels. We believe that our study will extend the horizons of tissue engineering, laying the groundwork for pioneering advances in controlled regenerative medicine and the future of bioprinting technology.

## 2 Materials and Methods

### 2.1 Materials

Graphene oxide (GO) was provided by Prof. Yuta Nishina (Okayama University, Japan). Reduced graphene oxide (rGO) was supplied by our collaborators. GP500 graphene nanoplatelets (GP) were acquired from GrapheneTech. Sodium alginate, calcium chloride (CaCl_2_), and phosphate buffer saline (PBS) were purchased from Sigma-Aldrich, USA. The 808 nm laser diode system was bought from Thorlabs (FPL808S). Polycaprolactone (PCL) was purchased from DomoTek, Spain. The culture medium used was Dulbecco’s modified Eagle medium (DMEM), acquired from Merck, and it was supplemented with 10% fetal bovine serum (FBS) from IBIAN Technologies and 1% penicillin/streptomycin solution from Merck. *E. coli*, Lysogeny Broth (LB), and LB agar (Miller) were purchased from Merck. Human fibroblast cells (hFBs) (C0135C) and the LIVE/DEAD Viability/Cytotoxicity Kit for mammalian cells (reference kit L3224) were bought from Thermo Fisher Scientific. The Cytotoxic 96@ Non-Radioactive kit was obtained from Promega (reference kit G1780).

### 2.2 Physicochemical Characterization of GMB

For Raman analyses, a Renishaw inVia Reflex Microscope at 532 nm with an incident power of 1% (1 mW μm^–2^) and a 100× objective was used to take at least 30 different measurements at different locations of the samples deposited on a silicon wafer. The thermogravimetric analyses (TGAs) of the freeze-dried dispersions of GO, rGO, and GP were carried out by using a TGA Q50 instrument (TA Instruments Company) from 40 to 800 °C with a ramp of 10 °C/min under N_2_ using a flow rate of 50 mL/min in platinum pans. The oxygen content of the samples was calculated from their elemental analyses (EAs), performed on an element analyzer (organic elemental analyzer UNICUBE from Elementar). The transmission electron microscopy (TEM) imaging was performed on a Zeiss EM 900 microscope (Carl Zeiss Microscopy GmbH, Germany) at 80 kV. The 10 μg/mL ethanol dispersions were sonicated for 10 s and drop-cast.

### 2.3 Photothermal Capacity

The GBM provided by the different suppliers were used to prepare dispersions at different concentrations in water: 2 and 0.5 mg/mL GO, 0.5 and 0.05 mg/mL GP, and 0.05 mg/mL rGO. Five hundred microliters of each dispersion was placed in an Eppendorf tube for irradiance tests, monitoring the temperature increase. Water was used as a control. Irradiations were performed inside an incubator at 37 °C. Solutions were irradiated from above for 10 min under 808 nm light (808 nm laser diode system from Thorlabs, FPL808S) with a power density of 1 W/cm^2^. A DomoBIO4A 3D bioprinter was ceded by DomoTek and used to print a PCL piece that was used as a support in the irradiation setup. A thermal camera FLIR ONE was used to determine the temperature of the dispersions every 30 s, and the heating performance was evaluated by subtracting the temperature at time zero.

The photothermal efficiency of the different GBM dispersions was determined following the protocol described by Feng et al. (see the Supporting Information).^37^ Briefly, 3 mL of each dispersion was prepared, transferred to crystal cuvettes, and irradiated under 808 nm light with a power density of 1 W/cm^2^. The temperature of the solution was constantly monitored by using a thermal camera. When a steady-state temperature was reached, the laser was turned off, and the temperature of the dispersions was registered every 30 s until they cooled down to room temperature. Before irradiation, the absorbance of every solution was measured at 808 nm with a Biowave II spectrophotometer (Biochrom, UK). The equations used are explained in the Supporting Information.

### 2.4 Bactericidal Capability of GBM Dispersions

To quantify the bactericidal capacity of the different GBM dispersions of both irradiated (808 nm, 10 min, power density of 0.5 W/cm^2^) and non-irradiated samples, the *E. coli* dispersion plate method was used.

First, *E. coli* cells were grown in Lysogeny Broth (LB) medium at 37 °C under 210 rpm shaking speed, and turbidity was adjusted to 1.9 × 10^5^ CFU/mL (O.D. was measured with a Biowave II spectrophotometer (Biochrom, UK) at 600 nm). Cells were collected by centrifugation, washed twice with PBS, and resuspended in the appropriate saline medium. *E. coli* cells were incubated with the different freshly prepared GBM dispersions in PBS at 37 °C at a shaking speed of 210 rpm for 2 h. The final concentrations were 0.5 and 0.05 mg/mL for GP and 0.05 mg/mL for rGO. A positive control consisting of bacteria harvested and incubated in the absence of any GBM was studied. Aliquots of the samples were withdrawn, diluted, and spread onto LB agar plates. After incubation at 37 °C, the capacity of the bacteria to form colonies was studied by comparing the resulting number of colony-forming units after each treatment. The increase in temperature during the irradiation was monitored using a thermal camera. All the treatments were performed at least in triplicate.

### 2.5 Cytotoxicity of GBM Dispersions

Human fibroblasts (6000 cells/well) were seeded and grown on a 96-well plate. Cells were incubated, and once they adhered to the surface of the wells, 100 μL of the different graphene dispersions was added to each well, and the cells were incubated at 37 °C. After 24 h of treatment, cell viability was studied by both LDH and Live/Dead assays following the protocols provided by the suppliers. Nontreated cells were used as the positive control. The absorbance was determined using a Synergy HTX Multi-Mode Microplate Reader (Winooski, VT, USA), and a Leica Thunder imaging system fluorescence microscope was used to visualize alive and dead cells. The images were analyzed by using ImageJ software.

### 2.6 Bioprinting of Hydrogels

Bioinks consisting of alginate, hFBs, and rGO were bioprinted by extrusion using the DomoBio4A bioprinter on loan from DomoTek S.L. Briefly, pregel mixtures were prepared by loading 0.45 mg of sodium alginate (9 wt %/vol), a cell suspension in culture medium supplemented with 10% FBS (157,000 hFB cells/mL pregel) and rGO at a final concentration of 200 μg/mL (Alg_rGO), into a bioprinting syringe. Control samples of alginate and hFBS without nanomaterial (Alg) were also prepared for comparison. Printing parameters were set using needles of 0.4 mm to fabricate 0.8 mm height structures of 4 layers. The geometry was designed as cylinders with a grid shape of 25% infill. After printing, 0.65 mL of a 0.2% CaCl_2_ solution was automatically added by centered and controlled drip by the bioprinter until the scaffold was covered entirely, and cross-linked hydrogels were instantly formed. The printability of both pregel mixtures (with and without rGO) was measured following the procedure described by Ouyang et al. (see the Supporting Information).^38^ Finally, the scaffolds were immersed in a growth medium supplemented with 10% FBS for further characterization.

### 2.7 Photothermal Properties and Biocompatibility of the Bioprinted Hydrogels

Scaffolds were irradiated at a constant temperature of 37 °C under 808 nm light for 10 min with a power density of 0.5 W/cm^2^. Non-irradiated hydrogels and scaffolds in the absence of rGO were used as controls. A thermal camera was used to check the rise in temperature of the bioprinted hydrogels. Subsequently, cell viability was studied by Live/Dead assay following the protocol provided by the supplier after irradiation (or not) of the samples after being incubated at 37 °C in a 5% CO_2_ incubator at 0, 24, and 72 h timepoints. A Leica Thunder imaging system fluorescence microscope was used to visualize live and dead cells, and the images were analyzed using ImageJ software.

## 3 Results and Discussion

### 3.1 Physicochemical Characterization of GBM

**Figure 1:**
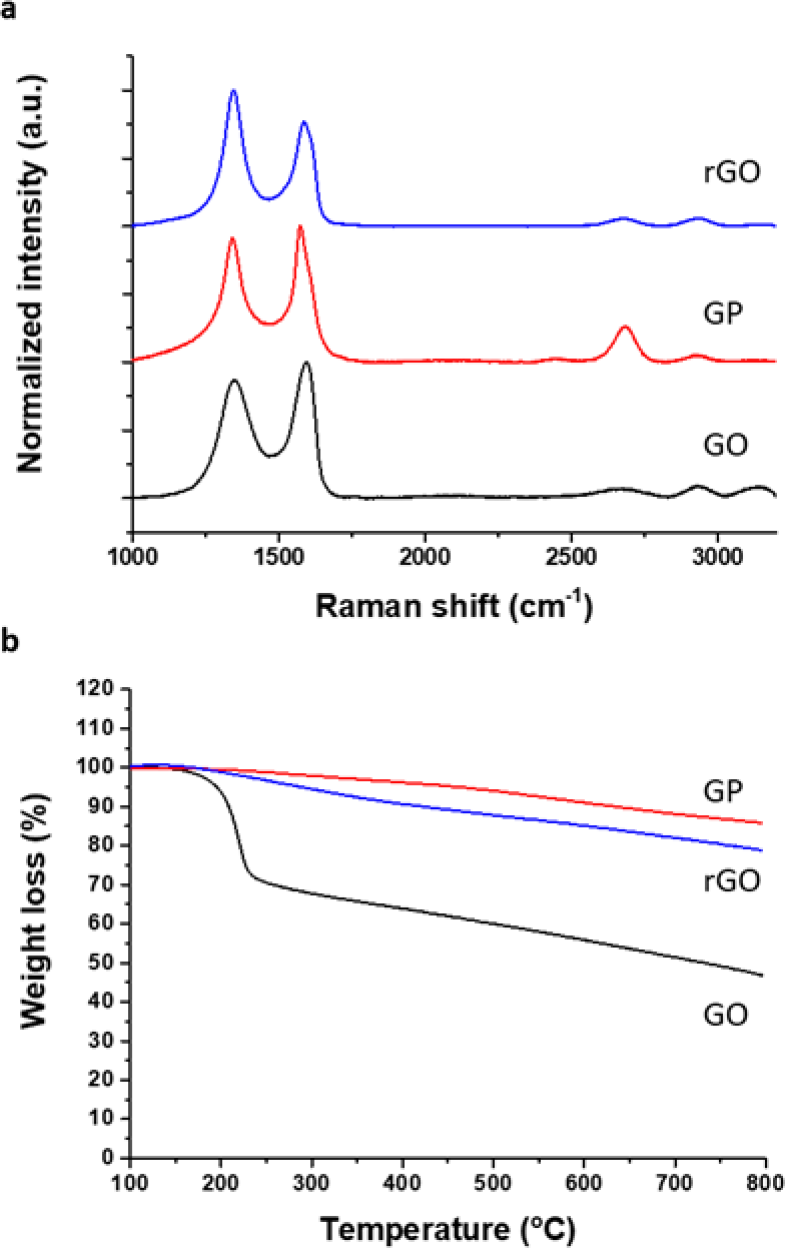
(a) Average Raman spectra and (b) thermogravimetric analyses of GO, GP, and rGO.

Three GBM were used, namely, graphene oxide (GO), reduced graphene oxide (rGO), and graphene nanoplatelet (GP). The average Raman spectrum of the three samples (Figure 1a) showed two intense peaks at ≈1580 cm^–1^ (G band) and ≈1350 cm^–1^ (D band), corresponding to the sp^2^ tangential mode and disordered carbon atoms, respectively.^39^ In addition, GP presents the 2D band at ≈2680 cm^–1^. The second-order scattering process generates this signal, suggesting in-plane vibrational modes of the sp^2^ carbon atoms in the GP lattices similar to those of pristine graphene.^40^ On the contrary, as expected, GO and rGO spectra display a bump instead of a clear 2D band.^41^ The average spectra of GP and GO have *I*(D)/*I*(G) ratios of 0.91 and 0.87, respectively, suggesting a lower level of disorder and defects on their lattices compared to rGO (*I*(D)/*I*(G) of 1.30). These defects may result from the reduction process carried out to obtain rGO, which can leave holes and irregularities in the graphene structure after removing oxygenated functional groups, as previously reported by different authors.^42,43^

TGA was performed to quantitatively assess the differences in chemical composition between the used graphene derivatives in terms of the functional groups present in the three materials (Figure 1b). At 800 °C, a weight loss of 54% was obtained for GO. In contrast, 15 and 22% weight loss values were obtained for GP and rGO, respectively. As expected, GO is the material presenting more oxygenated functional groups on its surface. These results indicate the low quantity of functional groups on the surface of GP and rGO resulting from the production process.

Elemental analysis yielded valuable information regarding GO, rGO, and GP composition. The oxygen contents were calculated from the ratios of C, H, N, and S (Table S1). GP and rGO exhibited carbon compositions exceeding 80% and oxygen contents of 12.80 and 18.70%, respectively. In contrast, GO is known to possess an oxygen content ranging between 40 and 50%, demonstrating the substantial presence of oxygenated functional groups on the surface of GO, which is consistent with earlier results obtained through Raman spectroscopy and TGAs.^44^ Representative GO, rGO, and GP flakes, each displaying prominent surface undulations, are shown in Figure S1. It becomes apparent that GO (with an approximate lateral size of 300 nm)^45,46^ and GP (featuring lateral sizes ranging from 200 to 400 nm) exhibit dimensions of relatively similar magnitude. In contrast, rGO flakes have larger lateral sizes (∼5 μm). Regarding the colloidal stability of the dispersions, GO and rGO flakes are less aggregated than GP sheets (Figure S2), as also confirmed by TEM analyses (Figure S1). It is important to note that while GO and rGO were provided as stable colloidal dispersions (10 and 5 mg/mL, respectively), GP was supplied in powdered form, challenging to disperse in water and tending to aggregate and settle.

### 3.2 Photothermal Capacity of GBM Dispersions

**Figure 2:**
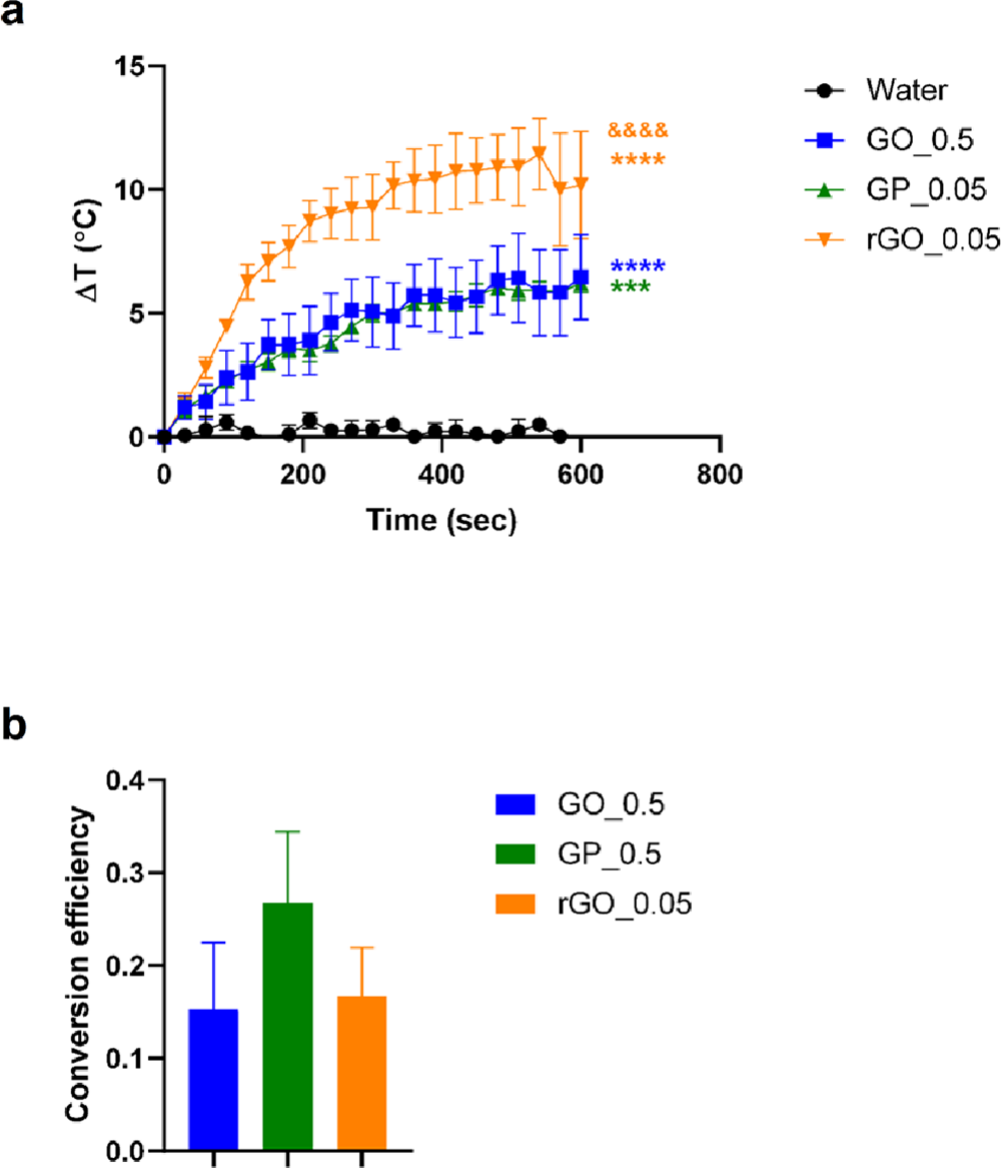
(a) Increase in temperature of GBM-containing dispersions under NIR irradiation (10 min, 808 nm, power density of 0.5 W/cm^2^): GO at 0.5 mg/mL (GO_0.5), GP at 0.05 mg/mL (GP_0.05), and rGO at 0.05 mg/mL (rGO_0.05). Water was used as a control. The results are expressed as average ± SEM for each time point (*n* = 4). Statistical analysis was performed using Tukey’s multiple comparisons test; the asterisk (*) denotes significant differences with respect to the control (*p* > 0.05, *p < 0.05, **p < 0.01, ***p < 0.001, ****p < 0.0001), and the ampersand (&) denotes significant difference among GBM (*p* > 0.05, ^&^*p* < 0.05, ^&&^*p* < 0.01, ^&&&^*p* < 0.001, ^&&&&^*p* < 0.001). (b) Photothermal conversion efficiencies for each GBM. The efficiency values were calculated following the protocol described by Wang et al. (see the Supporting Information).^28^

The photothermal capacity of GBM was evaluated in two different ways: First, we checked for each material’s capacity to generate heat by monitoring the increase in temperature of nanomaterial-containing dispersions under NIR irradiation. For comparison, GO was tested at 0.5 mg/mL (GO_0.5), GP was studied at 0.05 mg/mL (GP_0.05), and rGO was used at 0.05 mg/mL (rGO_0.05), while water was employed as the negative control. Temperature variations (Δ*T*) for each dispersion were determined using the images captured with a thermal camera. The results (Figure 2a) indicated that Δ*T* is material-, concentration-, and irradiation-dependent. In all instances, the rate of Δ*T* was most notable during the initial 2 min of irradiation, reaching a plateau in ∼5 min. GO_0.5 (Δ*T* = 6.5 °C, ****p < 0.0001) and GP_0.05 (Δ*T* = 6.2 °C, ***p < 0.001) exhibited the lowest Δ*T* despite the GO concentration being 10 times higher. Remarkably, by irradiation of rGO_0.05, we found the highest Δ*T* of 10.2 °C.

The photothermal efficiency of a nanomaterial refers to its ability to convert absorbed light into thermal energy due to its photothermal activity. A higher photothermal conversion efficiency implies a better thermal conductivity derived from a rapid cooling down after the laser was turned off.^37^ The results (Figure 2b) indicated that photothermal conversion efficiency strongly depends on the material where GO ≈ rGO < GP. By definition, the PTT efficiency is the ratio between the converted energy (measured by the Δ*T*) and the incident energy (given by the absorbed light), independent of the material concentration.^47^ Incorporating the elemental analysis and TGA outcomes, it is evident that GP boasts the lowest oxygen content within its graphene lattice. Generally, it is known that materials with higher reduction levels (high presence of sp^2^ C) display higher absorption in the NIR region and, therefore, bear higher PTT activity.^48^ Surprisingly, rGO exhibits greater photothermal activity despite having a higher oxygen content (than GP) within its structure, indicating that this effect cannot be attributed only to the chemical composition of the flakes but also to the colloidal stability. The latter likely hampers the application of GP, where although the PTT efficiency of the material is the highest among the others, the Δ*T* during irradiation is not the highest. Instead, while the PTT efficiency between GO and rGO is similar (ns, *p* > 0.05), there is a clear difference in Δ*T* performances. This latter fact can be due to the NIR light absorption that, at the same concentration, is much higher in the case of rGO, making it a better PTT agent.

### 3.3 Bactericidal Capability of GBM Dispersions upon NIR Irradiation

The *E. coli* dispersion plate method was employed to check for the bactericidal capacity, resulting from the PTT capacity of rGO_0.05, GP_0.05, and GP_0.5. As previously discussed, GO was excluded as a viable nanomaterial due to its insufficient photothermal capabilities. Irradiated and non-irradiated samples were included in the study to compare the intrinsic bactericidal capacity of the nanomaterials versus the bactericidal capacity derived from photothermal therapy.

**Figure 3:**
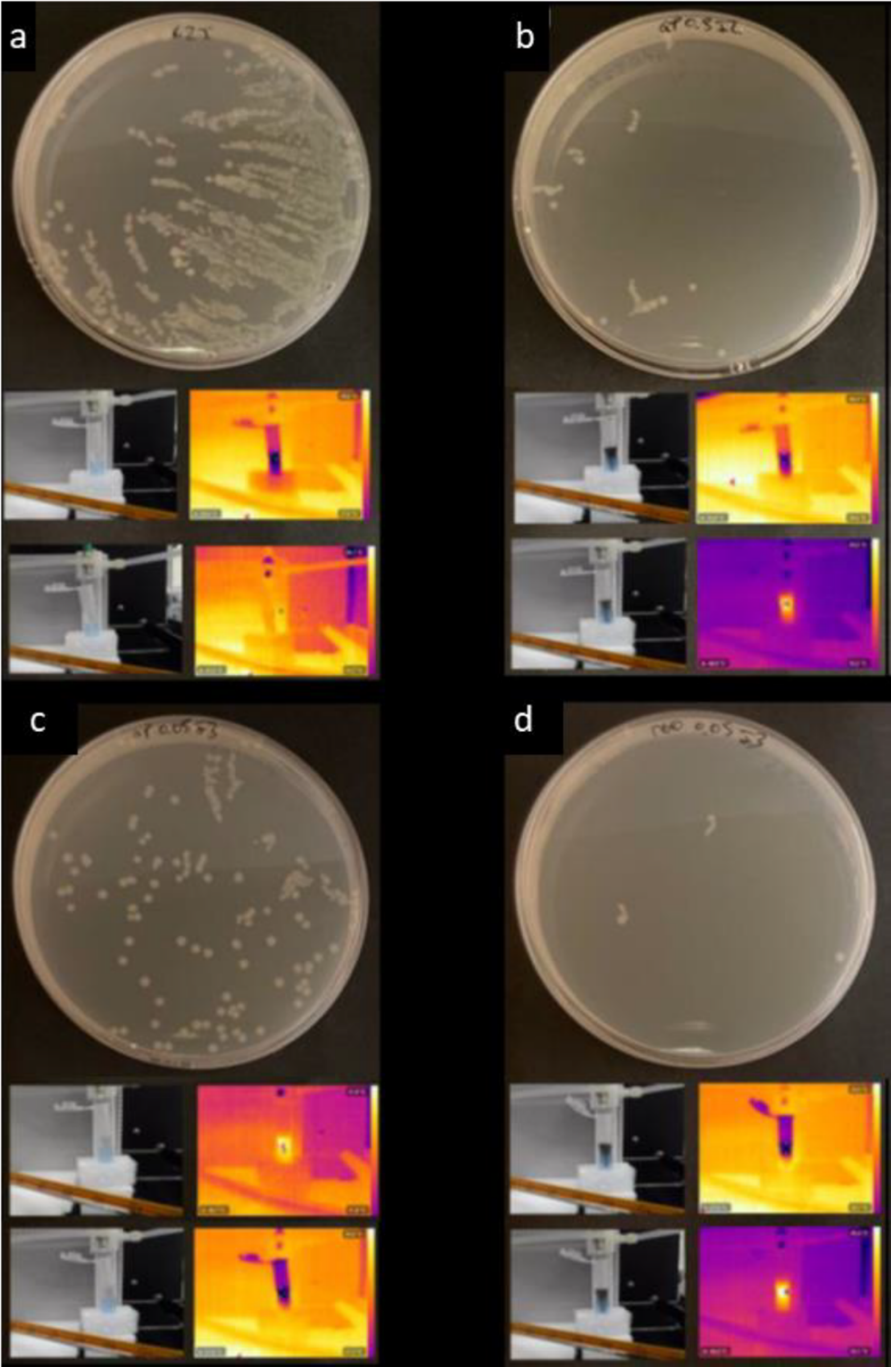
*E. coli* colonies formed after 24 h of incubation in the presence of irradiated samples (808 nm, power density of 0.5 W/cm^2^). (a) Control, (b) GP_0.5, (c) GP_0.05, and (d) rGO_0.05. Representative pictures taken with a thermal camera are displayed below each cultured dish: before irradiation (top images) and after 10 min of light stimulation (bottom photos).

Colonies were observed for each sample following 1 day of incubation without and with irradiation, and the antibacterial capacity was measured by quantifying the area of *E. coli* growth using the ImageJ program (Figure 3 and Figure S3, respectively). After irradiation, the control and GP_0.05 exhibited the slightest temperature increases, reaching 37 and 41 °C, respectively. These temperatures were insufficient to eliminate bacteria but facilitated their growth and propagation. Bacterial eradication became feasible when temperatures exceeding ∼45 °C were attained, as in the case of rGO_0.05 and GP_0.5, which achieved temperatures of ∼47 and ∼49 °C, respectively, effectively reducing the presence of *E. coli* colonies. Upon comparison, rGO showed the highest bacteria phototoxicity due to its high PTT activity at equal concentrations of all materials.

### 3.4 Cytotoxic Assays of GBM Dispersions

**Figure 4:**
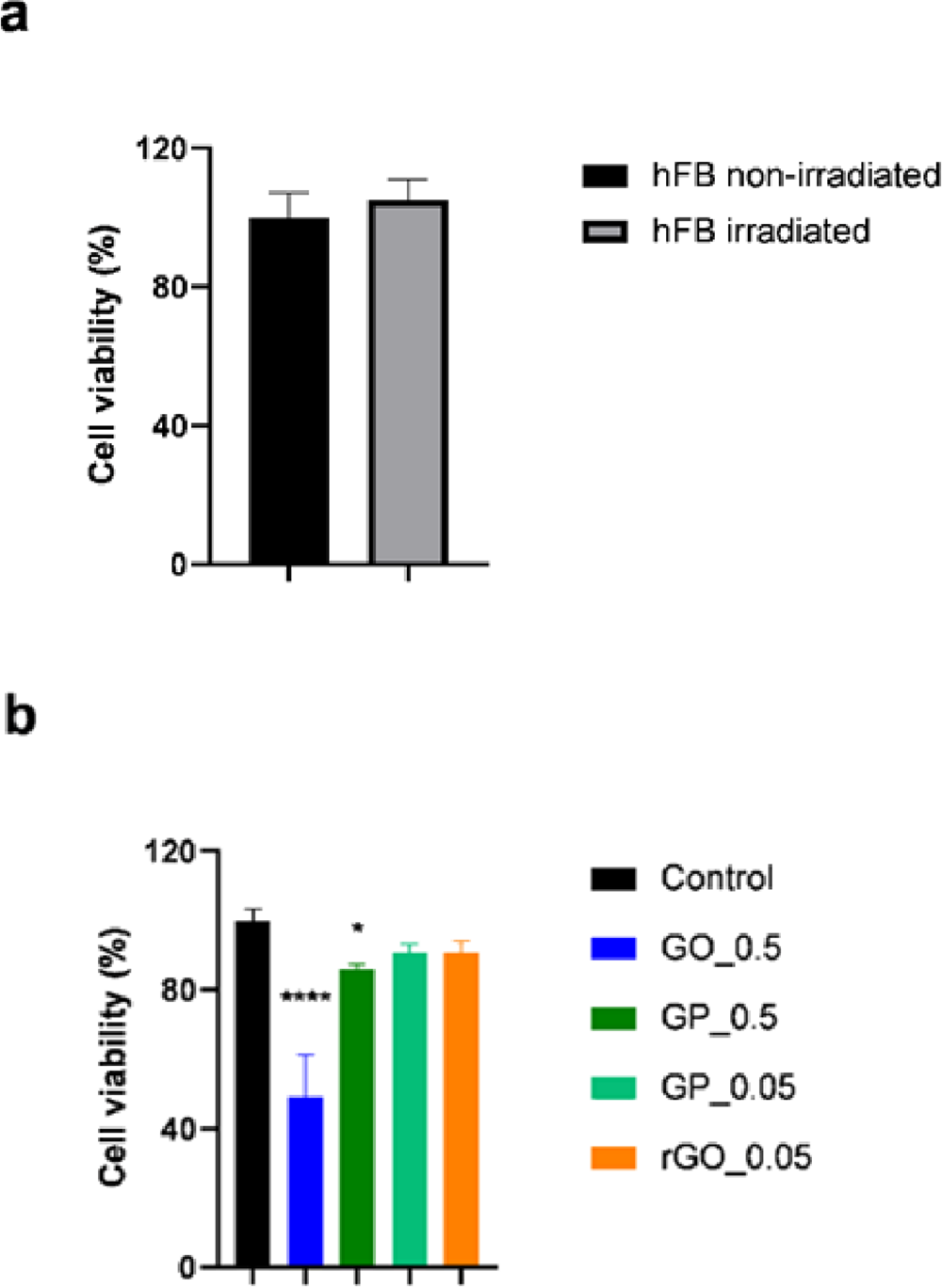
Cell viability of hFBs after 24 h of cell treatment in the presence of GBM measured by LDH assay. (a) Control experiment of the effect of the laser on hFB cell viability. (b) Cells treated with GO at 0.5 mg/mL (GO_0.5), GP at 0.5 mg/mL (GP_0.5), GP at 0.05 mg/mL (GP_0.05), and rGO at 0.05 mg/mL (rGO_0.05). Cells were irradiated with an NIR laser (10 min, 808 nm, power density of 0.5 W/cm^2^). The results are expressed as average ± SD for each time point (*n* = 4). Statistical analysis was performed using Tukey’s multiple comparisons test; the asterisk (*) denotes significant differences with respect to the control (*p* > 0.05, *p < 0.05, **p < 0.01, ***p < 0.001, ****p < 0.0001).

The intrinsic cytotoxicity of GBM was evaluated using human fibroblasts (hFBs) as in vitro models. Cell viability measured via LDH assay (Figure 4) revealed that GO_0.5 reduced hFB viability to 50%, indicating its cytotoxic effect, consistent with prior studies involving this nanomaterial showing that concentrations exceeding 50 μg/mL render GO toxic to fibroblasts.^49−51^ The toxicity of GO can be attributed to its substantial content of oxygenated functional groups,^41^ facilitating dispersion in water and cellular interactions, possibly leading to increased internalization and ROS stress production.^52^ Conversely, GP_0.5, GP_0.05, and rGO_0.05 exhibited cell viability values exceeding 80%, signifying their nontoxic nature and biocompatibility according to the ISO guidelines EN ISO 10993-5.^53^ The differences in these values were minimal, indicating that rGO and GP do not display toxicity to hFBs. Furthermore, LDH analysis was also conducted on previously irradiated fibroblasts to elucidate whether the irradiation conditions affect fibroblasts. The cell viability results demonstrated that hFBs remained unaffected by NIR light irradiation at the specified power density, resulting in a similar percentage viability (105 ± 6%) to the positive control (non-irradiated) sample (100 ± 7%). This fact opens the possibility of their future incorporation into smart hydrogels for biomedical applications.

Live/Dead analysis was also conducted to qualitatively assess and confirm the viability of hFBs in the presence of GBM. As depicted in Figure S4, the Live/Dead images were consistent with the results obtained from the previous LDH analysis. GO_0.5 exhibited the highest cell mortality, followed by GP_0.5. GP_0.05 and rGO_0.05 samples produced similar results to the control samples, indicating their nontoxicity to hFBs.

### 3.5 Printability, Photothermal Capacity, and Cell Viability Study of Alginate/rGO-Based Hydrogels

The remarkable performance of rGO, with significant temperature rise and excellent PTT efficiency at an exceptionally low concentration, also considering its noncytotoxic effect on hFBs, makes this nanomaterial an ideal candidate for synthesizing 4D-bioprinted smart hydrogels. The presence of rGO within the bioprinted network will provide the construct with controlled transformation features, i.e., heating upon light irradiation. Alginate is a popular bioink used in extrusion 3D bioprinting due to its cell-friendly properties and ability to undergo gelation.^54^ To showcase the potential of rGO, it was incorporated into alginate-based bioprinted hydrogels.

**Figure 5:**
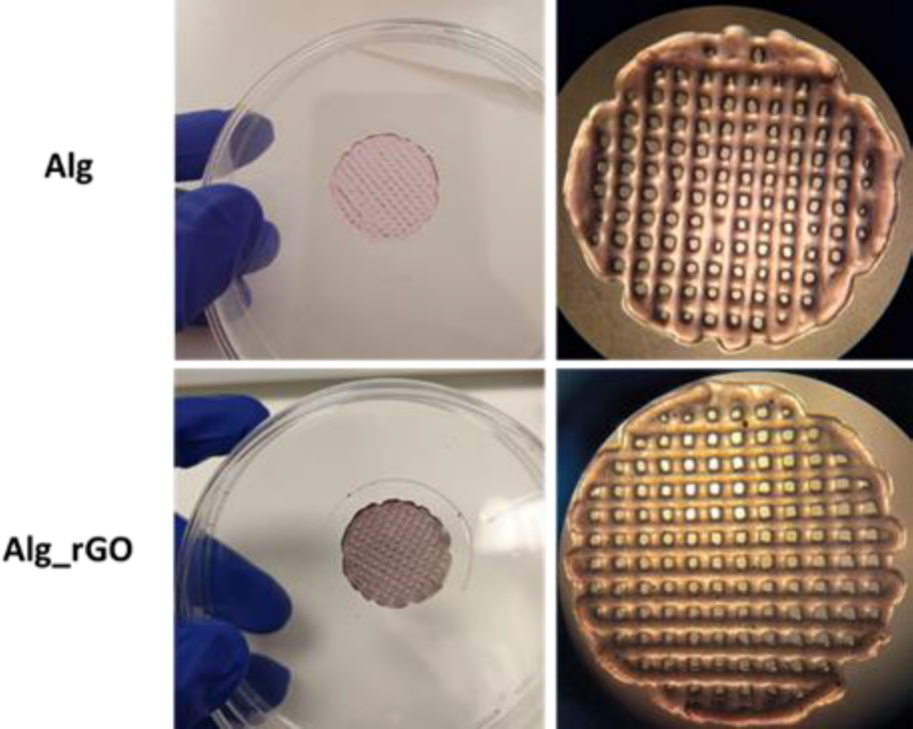
Digital images (left panel) and photographs under the compact multilens stereomicroscope (right panel) of bioprinted cylinders Alg (top) and Alg_rGO (bottom).

The bioink was prepared by dissolving alginate in a cell culture medium containing hFBs and rGO at a final concentration of 200 μg/mL (Alg_rGO). A control bioink without rGO was also prepared (Alg). The pregel mixtures were bioprinted using a DomoBio 4A bioprinter with printing parameters optimized to fabricate 0.8 mm-high structures of 4 layers. The geometry was designed as grid-shaped cylinders (Figure 5). After the bioprinting process, a 0.2% CaCl_2_ solution was automatically added for cross-linking the hydrogels. Bioink printability, namely, the ability to form a 3D structure with good fidelity and integrity, was measured for both pregel mixtures (with and without rGO),^38^ resulting in printability values of 0.95 ± 0.13 for Alg and 0.91 ± 0.05 for Alg_rGO. For an ideal gelation condition or perfect printability status, the interconnected channels of the constructs would demonstrate the square shape and printability values of 1.^38^ Therefore, our bioinks were perfectly bioprintable under the used conditions and, overall, rGO did not compromise the printability of the hydrogel, ensuring the successful fabrication of the constructs, as it was also predicted by visualizing the bioprinted cylinders under the compact multilens stereomicroscope (Figure 5).

Subsequently, the bioprinted scaffolds were irradiated with 808 nm light for 10 min at a power density of 0.5 W/cm^2^ to ensure that the PTT capability of the nanomaterial remained after intercalation into the hydrogel mesh. Bioprinted scaffolds in the absence of rGO were used as controls. Only Alg_rGO hydrogels locally warmed up ∼10 °C after irradiation, while Alg constructs did not evidence any response under external light stimulation (Figure S5).

**Figure 6:**
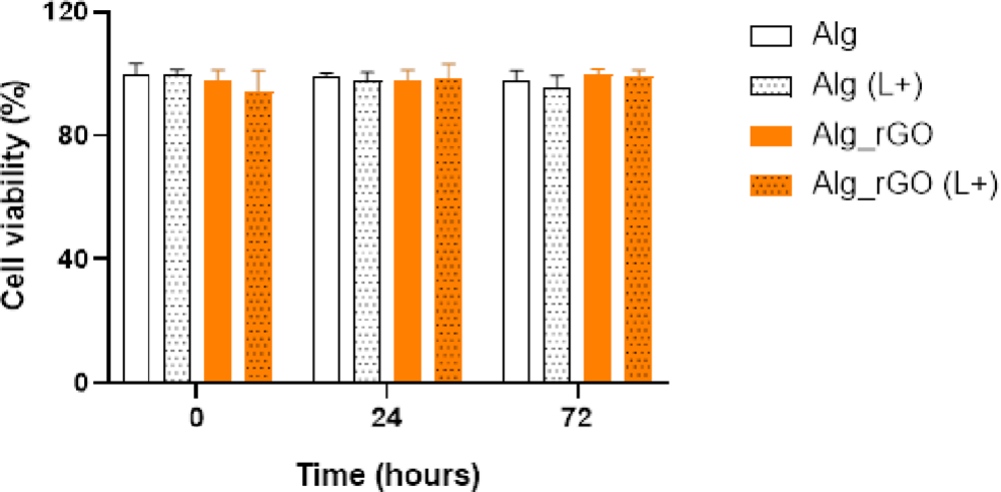
Viability of hFBs in the Alg (white columns) and Alg_rGO hydrogels (orange columns) at incubation timepoints of 0, 24, and 72 h after being irradiated (dashed columns) or not (solid columns).

Finally, to assess the cytotoxic effect of the rGO-containing 4D bioprinted smart hydrogels, the cross-linked scaffolds were immersed in a growth medium supplemented with 10% FBS, and cell viability was studied after 10 min NIR irradiation of the samples at 0, 24, and 72 h timepoints (Figure 6). Non-irradiated hydrogels and scaffolds without rGO were used as controls. Figures S6 and S7 contain representative images of Live/Dead experiments of hFBs embedded in Alg and Alg_rGO hydrogels, both irradiated and non-irradiated, respectively.

The results revealed that no significant differences in cell viability were observed due to the functionalization of the scaffold with Alg_rGO or the laser irradiation per se. It is widely known that the accuracy of printed structures plays a crucial role in creating functional tissues.^6^ Therefore, for bioinks to be useful for bioprinting, it is important to understand how printing forces impact the viability of enclosed cells. In this context, the rheological characterization of alginate-based bioinks has been widely studied to establish the influence of parameters such as the molecular weight of alginate in the viscosity and the final mechanical properties of the scaffolds.^55−57^

Moreover, alginate-based hydrogels are frequently combined with other materials to enhance their biocompatibility and suitability for 3D bioprinting applications. Even though sodium alginate-based hydrogel is a commonly utilized material for cell cultures, the inherent lack of cellular interaction properties in this polysaccharide often leads to its combination with other materials, such as gelatin^58^ or cellulose.^59^ Different works report on using graphene-based materials and alginate to prepare bioinks for wound dressing,^60−62^ proving the enhanced printability and mechanical properties of the final scaffolds, however, without including cells into the ink formulation.

Here, the challenges were addressed at hand without the necessity of introducing supplementary polymers, only incorporating rGO at a low concentration (200 μg/mL), which not only alleviated the issue but also endowed the construct with photothermal capabilities, thus rendering Alg_rGO as smart material suitable for photoactivatable biomedical applications, since the irradiation conditions used did not compromise hFB viability. We acknowledge that there is scarce information on the possible toxicity of graphene-based material through skin exposure, and there is still a long way to go before their safe use in the bioengineering field.^63^ In addition, very recently, it was reported that graphene nanoplatelets do not induce any sensitization or irritation in vivo.^64^ In our study, the high biocompatibility of Alg_rGO, even during irradiation, makes rGO a promising candidate for further studies in bioink formulations.

## 4 Conclusions

Our study involved the physicochemical characterization of three distinct GBM, GO, GP, and rGO, correlating their structural features with photothermal capabilities as photoactive skin-inspired dressings. The photothermal capacity of the three GBM was assessed, and the results indicated that GP and rGO displayed remarkable photothermal efficiency, with rGO outperforming the others despite its lower concentration. In contrast, GO exhibited lower photothermal activity and higher cytotoxicity on hFBs. The superior photothermal efficiency of rGO is probably due to its physicochemical properties and better dispersibility in aqueous suspension, thereby designating it as the primary candidate for subsequent experiments.

As a proof of concept, rGO was integrated into alginate-based hydrogels and successfully 4D-bioprinted since the inclusion of rGO does not compromise the printability of the hydrogel, ensuring the successful fabrication of intricate and complex constructs and providing the bioprinted scaffolds with heating capability after laser irradiation. The resulting smart bioprinted hydrogels demonstrated photothermal capacity, even when using only 200 μg/mL rGO. Moreover, cell viability studies have shown that the irradiation conditions or the presence of Alg_rGO in the hydrogel did not compromise hFB viability over time.

In conclusion, combining rGO with alginate-based hydrogels showed promise in developing smart materials without additional polymers for photoactivatable biomedical applications. This approach addresses the challenges associated with conventional material-only printing techniques. It holds potential for further research and development, especially considering using Alg_rGO constructs for biomedical applications in bioprinting and tissue engineering. Future studies are warranted investigating phenotypic changes of dermal fibroblasts and the production of ECM components in more complex coculture studies using keratinocytes. Furthermore, photobiomodulation induced by NIR irradiation should be included in studies involving photoactive materials.

## Supporting Information Available

Determination of photothermal efficiency values, semiquantification of printability, representative TEM images of GO, rGO, and GP nanomaterials, digital pictures of nanomaterials’ dispersions at different concentrations, *E. coli* colonies formed and bacterial viability quantification, Live/Dead analysis, and representative images of bioprinted scaffolds before and after irradiation (PDF)
