# Supplementary material for: Light-Responsive and Antibacterial Graphenic Materials as a Holistic Approach to Tissue Engineering
Source: ACS Nanosci Au. 2024 Jun 7;4(4):263–72. doi: 10.1021/acsnanoscienceau.4c00006 (PMC11342345; doi:10.1021/acsnanoscienceau.4c00006)
Supplement: Supplementary file 1 — ng4c00006_si_001.pdf [file ng4c00006_si_001.pdf]

Supporting Information

Light-Responsive and Antibacterial Graphenic Materials as a Holistic Approach to Tissue Engineering.

Andrea Ferreras [#], Ana Matesanz [Δ], Jabier Mendizabal [¥], Koldo Artola [¥], Yuta Nishina [∞,£], Pablo Acedo [Δ], José L. Jorcano [#,≠], Amalia Ruiz [&,*], Giacomo Reina [§,*], Cristina Martín [#,*].

[#] Department of Bioengineering, Universidad Carlos III de Madrid, Leganés 28911, Spain.

[Δ] Department of Electronic Technology, Universidad Carlos III de Madrid, Leganés 28911, Spain.

[¥] Domotek ingeniería prototipado y formación S.L., San Sebastián, 20003 Spain.

[∞] Graduate School of Natural Science and Technology, Okayama University, Okayama 700-8530, Japan.

[£] Research Core for Interdisciplinary Sciences, Okayama University, Okayama 700-8530, Japan.

[≠] Instituto de Investigación Sanitaria Gregorio Marañón, Madrid 28007, Spain

[&] Institute of Cancer Therapeutics, School of Pharmacy and Medical Sciences, Faculty of Life Sciences, University of Bradford, Bradford BD7 1DP, United Kingdom.

[§] Empa Swiss Federal Laboratories for Materials Science and Technology, St. Gallen 9014, Switzerland.

Determination of photothermal efficiency values

The efficiency values were calculated by following the protocol described by Feng et al.[28]

η = $[h(Tmax - Tsur)]{-}Qs$ / $[I(1{-}10{-}A\lambda)]$ (1)

Being η the photothermal conversion efficiency, h the heat transfer coefficient, S the surface area of the sample cuvette, $Tmax$ the steady-state temperature, $Tsur$ the temperature of the surrounding, Qs the heat associated with the light absorbance of the solution, I the incident laser power, and $A\lambda$ the absorbance of the nanomaterials at a wavelength of 808 nm. Qs is defined through the following equation (2):

Qs = $(mDcD\Delta T)$ / t (2)

Where mD is the mass of the water solution, cD the water heat capacity, ΔT the increase in water temperature, and t the duration of the irradiation. hS, which can be named as θ, is defined as follows (3):

θ = $(T - Tsur)$ / $(Tmax - Tsur)$ (3)

To solve for θ, a sample time constant τs is defined (4):

τs = $(\Sigma i\ mi\ cp,)$ / hS (4)

Also, as reported in the literature[28], the following relation can be established (5):

$t = -\tau s ln\theta$ (5)

Therefore, the time constant is obtained from the equation of the graph when plotting time data vs $ln\theta$. hS can be defined according to the obtained τs, also considering the mass of the solution and the heat capacity of water.

Semi-quantification of printability

The printability values were calculated by following the protocol described by L Ouyang et al.[29]

When the bioink gels ideally, the extruded filament shows a smooth, consistently sized morphology, forming regular grids and square holes in the constructs. In contrast, under-gelation leads to a more liquid-like state, causing the upper layer to merge with the lower layer and creating roughly circular holes in the process. It is known that circularity (*C*) of an enclosed area is defined as:

$$C = (4\pi A)/L^2$$

where, L means perimeter and A means area. Circles have the highest circularity ($C = 1$)

If the C value approaches 1, the shape is more circular. Circularity for a square shape is π/4. We establish bioink printability (*Pr*) for a square shape using the following function:

$$Pr = \pi/(4 \cdot C) = L^2/(16 \cdot A)$$

Under ideal gelation or perfect printability, the interconnected channels in constructs exhibit a square shape, with a *Pr* value of 1. A higher *Pr* value indicates a greater bioink gelation degree, while a lower *Pr* value suggests a smaller gelation degree. The *Pr* value for each bioprinted scaffold was determined by analyzing optical images in ImageJ software to calculate the perimeter and area of interconnected channels (n = 3).

Table S1. Elemental analyses of rGO and GP.

**GBM**	**ELEMENTAL ANALYSIS ± SD (%wt)**				
	C	H	N	S	O
rGO	80.32 ± 0.74	0.69 ± 0.09	0.027 ± 0.02	0.26 ± 0.04	18.70
GP	86.31 ± 0.42	0.37 ± 0.04	0.48 ± 0.005	0.028 ± 0.01	12.80

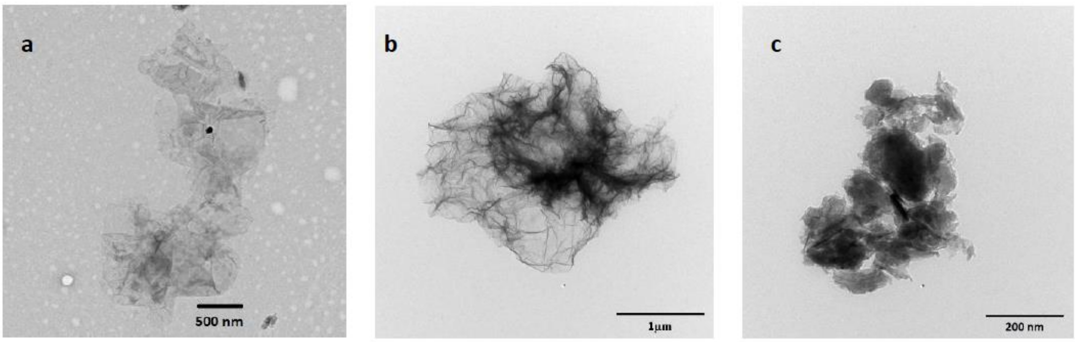

Figure S1. Representative TEM images of a) GO, b) rGO and c) GP nanomaterials.

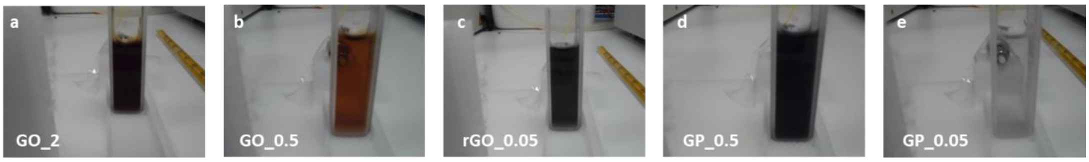

Figure S2. Digital pictures of a) GO_2, b) GO_0.5, c) rGO_0.05, d) GP_0.5 and e) GP_0.05 dispersions.

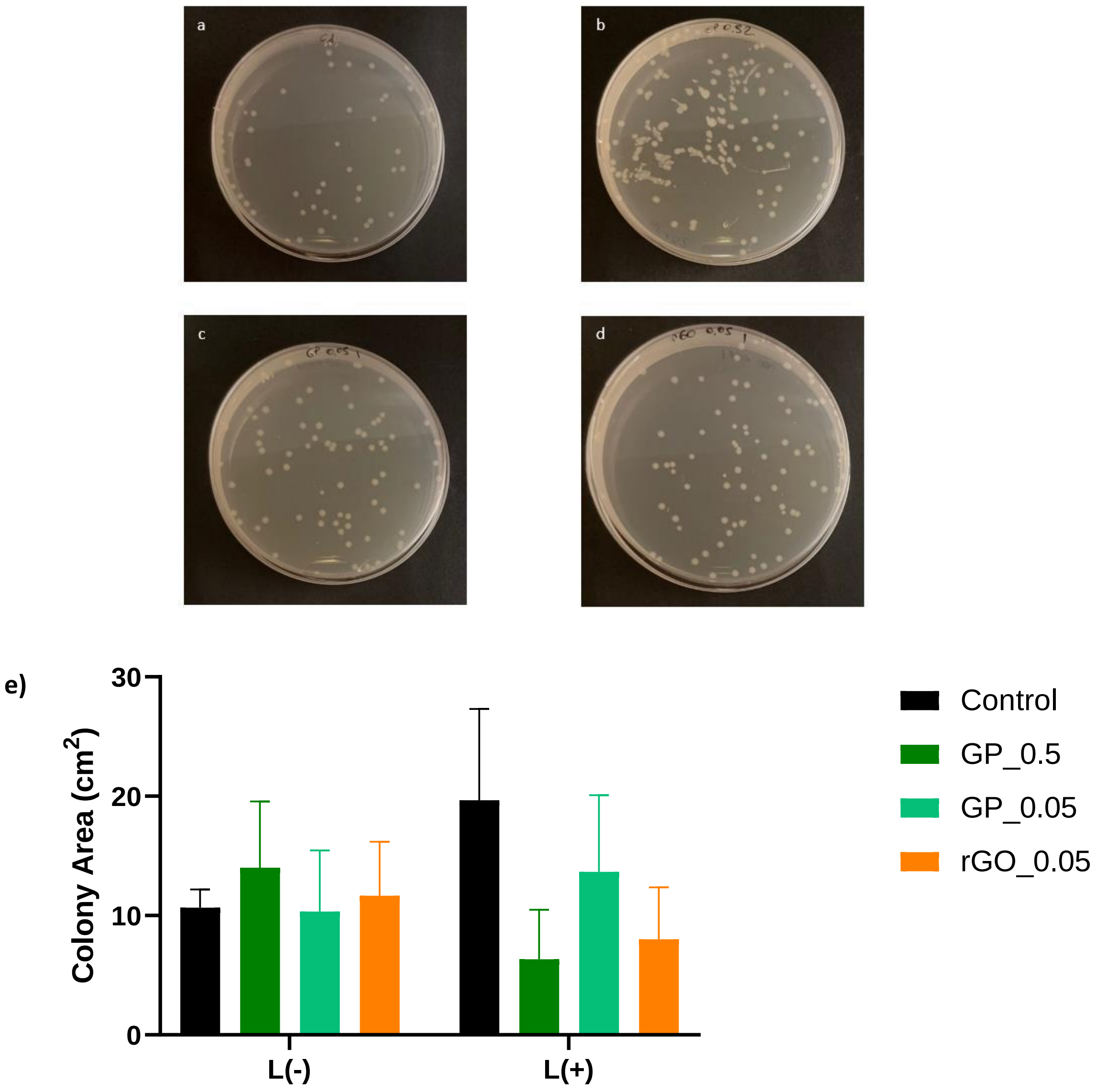

Figure S3. *E. coli* colonies formed after 24 h of incubation in presence of unirradiated samples. a) Control, b) GP_0.5, c) GP_0.05 and d) rGO_0.05. e) Bacterial viability quantified as the area of *E. coli* (%) grown on culture plates of unirradiated, L(-), and irradiated L(+) samples.

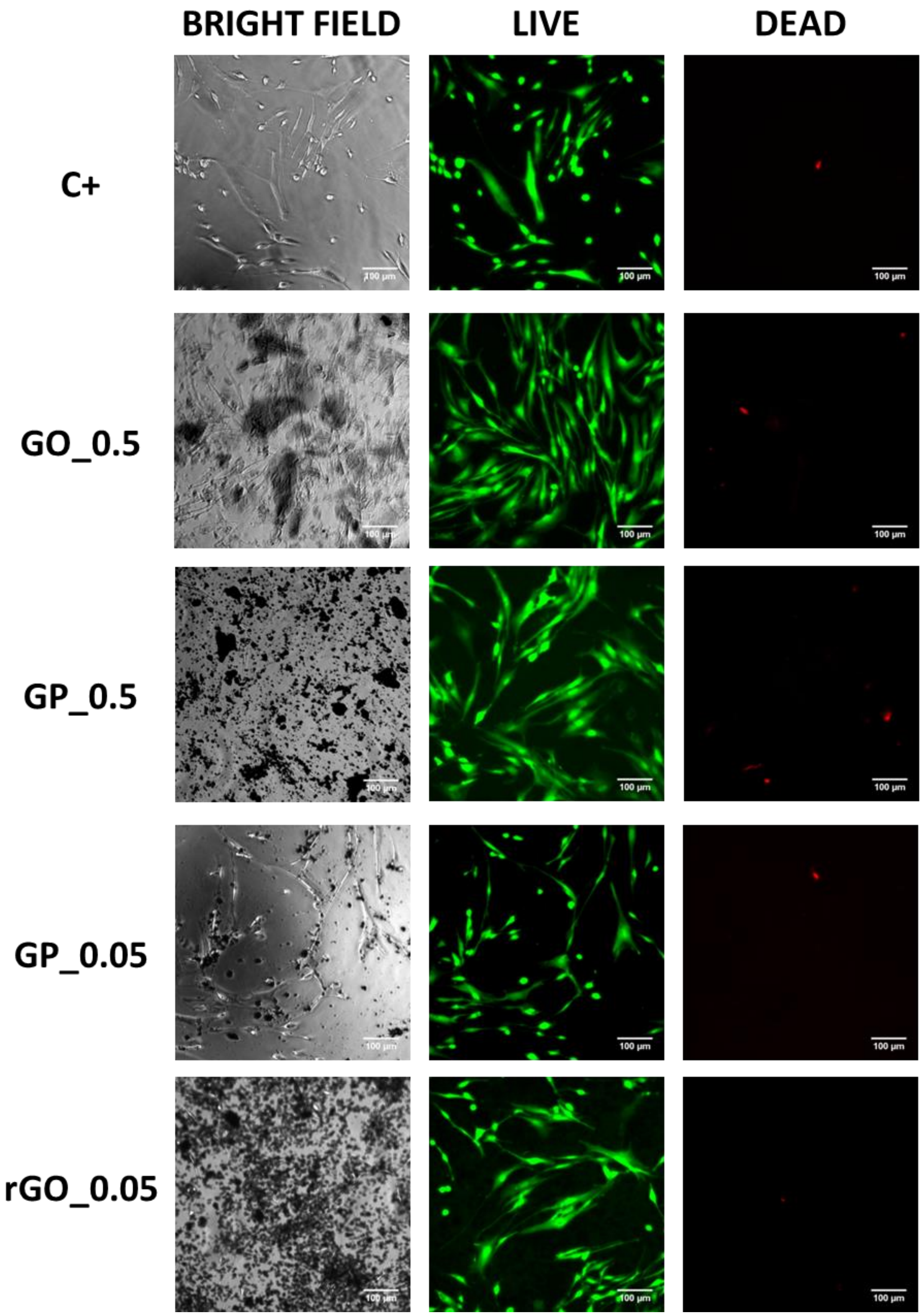

Figure S4. Live/Dead analysis, after exposure to the GBMs at different concentrations. Scale bars: 100 µm.

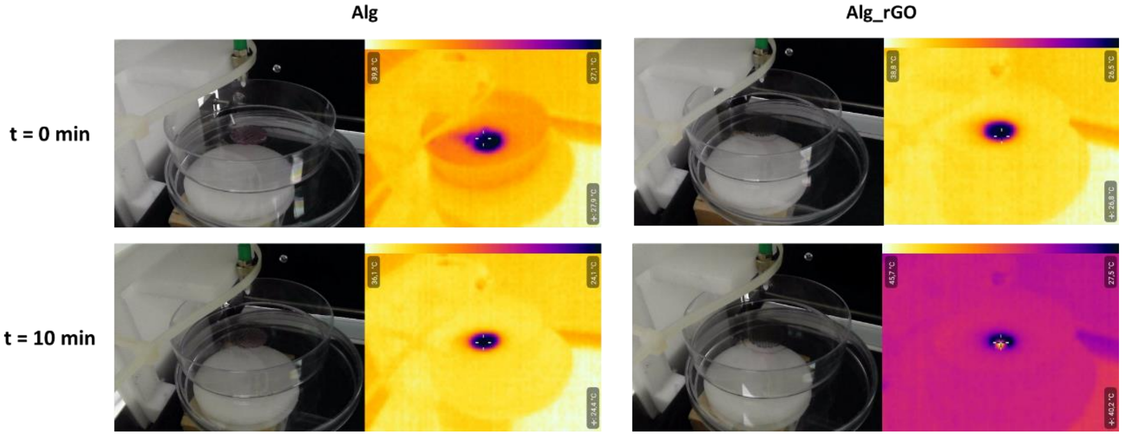

Figure S5. Representative images of bioprinted scaffolds Alg (left panel) and Alg_rGO (right panel) before irradiation (top panel) and after irradiation with 808 nm light for 10 min at a power density of 0.5 W/cm^2 (bottom panel).

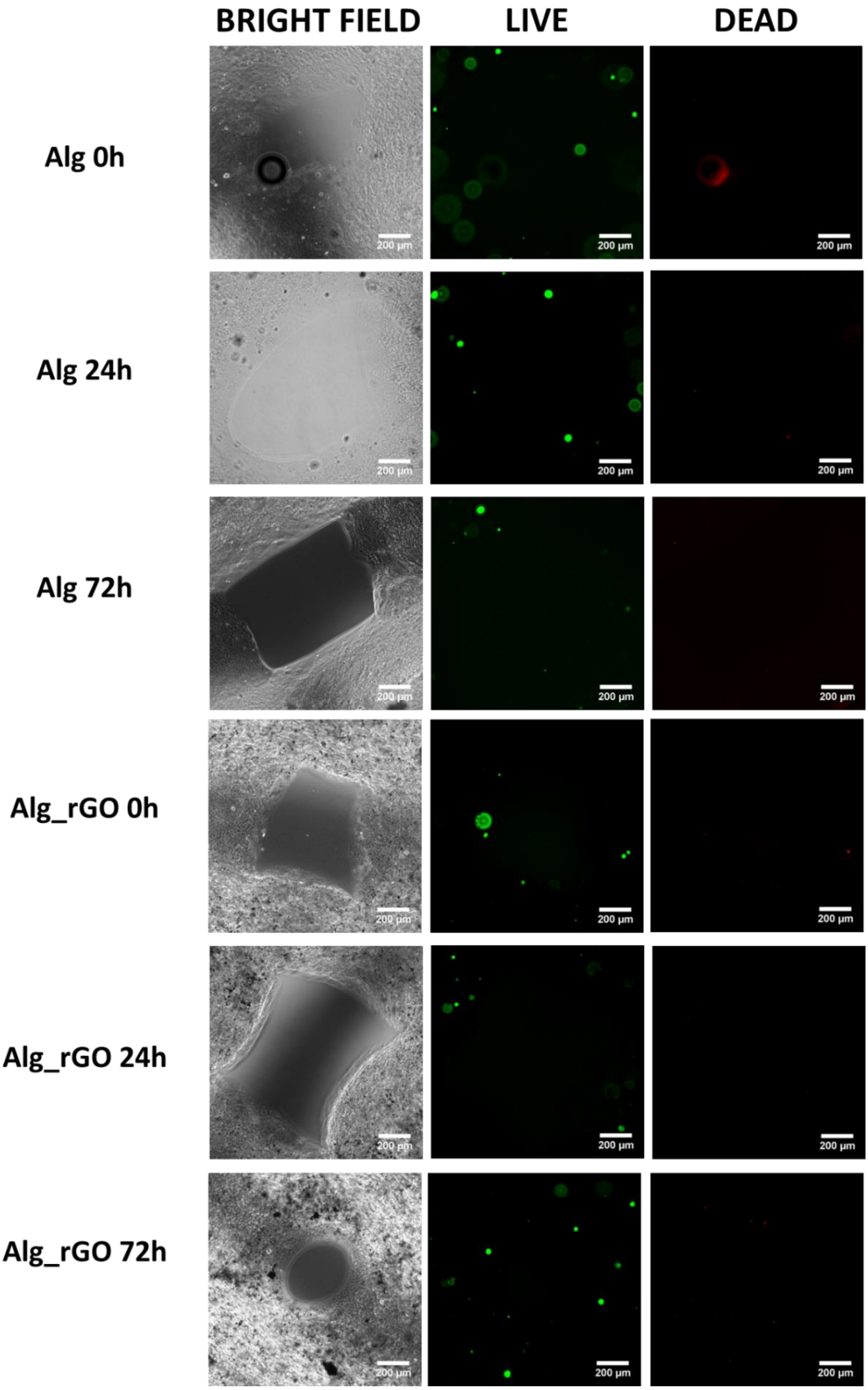

Figure S6. Representative pictures from the Live/Dead experiments of hFBs embedded into non-irradiated Alg and Alg_rGO hydrogels, at incubation time points of 0 h, 24 h and 72 h.

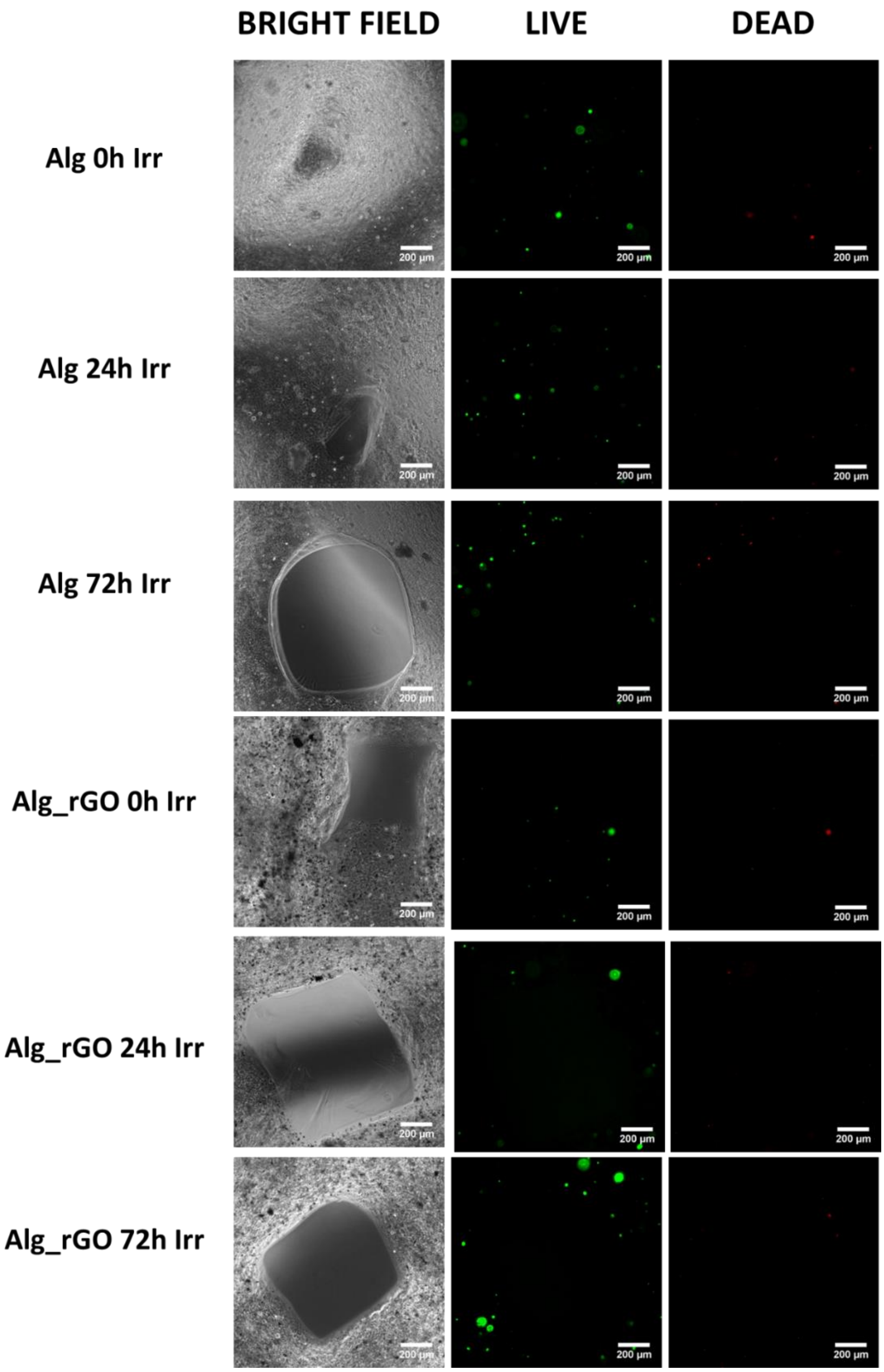

Figure S7. Representative pictures from the Live/Dead experiments of hFBs embedded into irradiated Alg and Alg_rGO hydrogels, at incubation time points of 0 h, 24 h and 72 h.
